# Effect of Ilioinguinal Neurectomy on Chronic Pain following Herniorrhaphy

**DOI:** 10.5812/traumamon.6581

**Published:** 2012-10-10

**Authors:** Hadi Khoshmohabat, Farzad Panahi, Ali Akbar Alvandi, Shaban Mehrvarz, Hasan Ali Mohebi, Ehsan Shams Koushki

**Affiliations:** 1 Department of Surgery, Trauma Research Center, Baqiyatallah University of Medical Sciences,, Tehran, IR Iran; 2 Department of Surgery, Faculty of Medicine, Baqiyatallah University of Medical Sciences, Tehran, IR Iran

**Keywords:** Neuralgia, Hernia, Inguinal, Hypoesthesia, Neurectomy, Liechtenstein

## Abstract

**Background:**

Inguinal hernia is one of the most common male diseases all over the world with an incidence rate of 18-24% throughout life. Chronic inguinal pain is one of the complications that prolong return to work time.

**Objectives:**

The main aim of this study was to determine the effect of ilioinguinal neurectomy on postoperative chronic pain (PCP) in patients that underwent open inguinal hernia repair via the Lichtenstein method.

**Materials and Methods:**

In this randomised controlled clinical trial, male patients with unilateral inguinal hernia were randomized into two groups: 74 cases in the preserved-nerve group and 66 cases in the nerve-excised group. The method of herniorrhaphy was the classic Lichtenstein method. Pain and numbness were evaluated at 1 day, 1 week, 1 month, 6 months and 1 year after surgery via visual analogue scale (VAS) system. We used SPSS ver.16 for analysis.

**Results:**

All patients were male with mean age of 39.1 years (with a range of 18 to 68 years). The follow-up rate was 100% after 1 year. Pain severity was significantly lower in nerve-excised patients at 1 day, 1week, 1 month and 6 months after surgery; but it was not significant after one year, although overall pain severity was low. Numbness was significantly higher in excised patients at all endpoints (1 day, 1month, 3 months, 6 months and one year after surgery).

**Conclusions:**

Ilioinguinal nerve excision at the time of inguinal hernia repair decreased post-surgical inguinal pain, and it can be used as a routine method in herniorrhaphy.

## 1. Background

Chronic post-herniorraphy pain is among the most frequent complications after inguinal hernia repair. In some patients it can be disabling. The prevalence of postoperative chronic pain (PCP) ranges from 0 to 63%, regardless of the type of hernia repair and may be secondary to a multitude of disorders including the post-herniorrhaphy pain complex syndrome (1). The ilioinguinal nerve innervates the mons pubis and inguinal crease together with a very anteroproximal part of the root of the penis or labia majora. The described patterns of innervation were bilaterally symmetric in 40.6 percent of cadavers (2).

Nociceptive or neuropathic pain syndromes related to hernia surgery have to be differentiated from other sources (1). Chronic pain following inguinal hernia repair is becoming a significant clinical problem, as shown by the rising number of publications over the last 10 years dealing with the postoperative pain syndrome (3). First of all, it is important to differentiate chronic pain from acute pain. Early postoperative pain is usually seen soon after surgery; it is easily and successfully managed with analgesics and generally resolves within 15 to 30 days of surgery without the need of further treatment. On the contrary, chronic pain, generally seen 3 months after surgery may result in a potentially debilitating condition. And sometimes resulting in the patient being unable to perform daily activities or to return to work (3). Various patterns of ilioinguinal or iliohypogastric nerve injury during elective inguinal hernia have been recognized: inadvertent suture entrapment, partial division, crushing, or diathermy burns; the end result is the occurrence of serious, persistent postoperative pain and occasionally can be responsible for significant patient debilitation (4). Postoperative neuralgia is further augmented as a complication for the surgeon by the accompanying risk of litigation. In fact, 5% to 7% of patients who experience postherniorraphy neuralgia will sue their surgeons (5). In another study the incidence of chronic pain after inguinal hernia has been estimated to be between 1% and 19% (6); 6% of patients with repaired inguinal hernia suffer from severe PCP, and groin pain prevents the patients from carrying out their daily activities. In order to reduce the incidence of PCP, elective neurectomies have been employed; however, the effectiveness of neurectomy is controversial (1). Because nerve excision eliminates postsurgical pain caused by entrapment, inflammation, or fibrotic reactions around the nerve, there seem to be some theoretic benefit of this practice (7). The neuropathic groin pain after inguinal hernia repair is usually due to a neuroma of the ilioinguinal, iliohypogastric, or genitofemoral nerve. Although the surgical approach to the ilioinguinal nerve is now well established, it has been difficult to identify the genitofemoral nerve reliably enough (8).

The underlying causes of PCP include neuropathic and non-neuropathic conditions. The neuropathic causes are nerve compression by perineural fibrosis, suture, staples, prosthetic material and nerve injury during surgery. Walking and hyperextension of the hip trigger this type of pain. The non-neuropathic (somatic pain) causes are periosteal reaction, scar tissue, mechanical pressure of folded mesh called ‘meshoma’ and visceral pain (encountered only on ejaculation); Neuropathic causes, especially chronic nerve irritation, are considered as an important cause of moderate or severe PCP (1). In a metaanalysis about groin hernia repair with synthetic mesh, the overall incidence of persistent pain was 5% (9). The identification and preservation of nerves during surgery reduce the risk of PCP; Another factor that affects PCP is the structure of the mesh and randomized clinical studies showed that a lightweight mesh may cause less PCP than a heavyweight mesh (10). Theoretically, excision of the ilioinguinal nerve would eliminate the possibility of postoperative neuralgia arising from entrapment, inflammation, neuroma, or fibrotic reactions (5).

Non-identification of the ilioinguinal nerve during open inguinal mesh repair carries a significant risk for the development of pain lasting longer than 3 months (11). The ilioinguinal nerve is the most at risk for entrapment because it lies immediately beneath the divided external oblique fascia and can be entangled in sutures used for the hernia repair and to reapproximate the external oblique fascia (12).

## 2. Objectives

The main aim of this study was to evaluate long-term and short-term outcomes (pain, numbness and complications) following ilioinguinal nerve-excision compared to nerve-preservation.

## 3. Material and Methods

This was a randomized controlled clinical trial on male patients with uni-lateral inguinal hernia admitted to our hospital. Informed consent was obtained before surgery from hospital-admitted patients. Patients were randomized in two groups: 74 cases in preserved-nerve group and 66 cases in nerve-excised group. Pain and numbness were evaluated at 1 day, 1 week, 1 month, 6 months and 1 year after surgery with VAS system. We used SPSS ver.16 for analysis. Pain and numbness was assessed before the operation and on days 0, 1, 7, and 6 months and 1 year postoperation.

Between june 2009 to july 2010 patients consecutively diagnosed with primary unilateral inguinal hernia and scheduled for Lichtenstein’s operation were included; 140 patients older than 18 years who suffered from primary inguinal hernia were randomized into two clinically comparable groups: 66 patients were in the nerve-excision group (group A), and 74 patients were in the nervepreservation group (group B). In group A, we performed Lichtenstein hernia repair and neurectomy; in group B, only Lichtenstein’s operation was performed. Patients with recurrent, bilateral, strangulated and incarcerated hernias and patients with history of diabetes mellitus, history of previous abdominal operation, opium addiction and cerebrovascular accident were excluded.

Informed consent, including explanation of the risk of chronic pain, was obtained from all patients in an outpatient setting. We explained probable complications to all patients. Analyzed parameters included age, gender, preoperative pain, post-operative pain, post-surgical complications (surgical site infection, hematoma, testicular ecchymosis, tingling in the site of surgery), physical activities, type of hernia (direct or indirect) and type of anesthesia. The primary outcome was the incidence of disabling pain and numbness at 6 months and 1 year post-surgery. Also surgical incision length on the first day after surgery was measured with a ruler. And returnto-work time was recorded. Comparative data about the groups are shown in Tables 1, 2, 3.

**Table 1:** Comparison of Pain Severity at Different Times after Surgery Between the Nerve-excision Group and Nerve-preservation Group

Follow up times	Nerve-excision group	Nerve-preserved group	*P*
**A day after surgery**	7.67 ± 0.7	8.5 ± 0.6	0.000
**A week after surgery**	2.03 ± 0.7	3.72 ± 0.7	0.000
**A month after surgery**	0.56 ± 0.5	1.89 ± 0.6	0.000
**Six months after surgery**	0.33 ± 0.4	0.91 ± 0.6	0.000
**One year after surgery**	0.15 ± 0.3	0.22 ± 0.4	< 0.05

**Table 2:** Comparison of Numbness Severity at Different Times After Surgery Between the Nerve-excision Group and Nerve-preservation Group

Follow up times	Nerve-excision group	Nerve-preserved group	*P*
**A day after surgery**	0.41 ± 0.4	0.19 ± 0.3	0.000
**A week after surgery**	2.86 ± 0.7	1.31 ± 0.5	0.000
**A month after surgery**	4.61 ± 0.7	1.91 ± 0.6	0.000
**Six months after surgery**	5.8 ± 0.7	2.15 ± 0.7	0.000
**One year after surgery**	7.52 ± 0.7	1.3 ± 0.7	0.000

**Table 3:** Comparison of Post-surgical Complications Between the Nerve-excision Group and Nerve-preservation Group

Complications	Nerve-excision group, %	Nerve-preserved group, %
**Infection**	1, 7.7	2, 8.3
**Hematoma**	1, 7.7	5, 20.8
**Seroma**	2, 15.4	4, 16.7
**Testicular Edema**	4, 30.8	5, 20.8
**Tingling**	5, 38.5	8, 33.3
**Total**	13, 100	24, 100

This project was approved by the University Research Council and by the local ethics committee. We performed ilioinguinal neurectomy in this study because this nerve always lies between the mesh and the muscular layer after anterior mesh repair, and fibrous tissue can cause nerve entrapment. In this study, the inguinal hernia of the patient was repaired using the open tensionfree mesh technique as described by Lichtenstein et al. Surgical technique was the same in both groups, and surgery was performed with the patient under spinal and general anesthesia. Local anesthetics were not used. Postsurgical analgesic medications were opiods in the early postsurgical hours and then with nonsteroidal antiinflammatory drugs.

Hernia repairs were performed by two experienced surgeons. In group B, tension-free hernia repair was performed and Polypropylene of 7×15 cm in size was secured to the floor of the inguinal canal by 2/0 nylon stitches. The ilioinguinal nerve mentioned above was identified and preserved. Then, the external oblique fascia was closed above the spermatic cord, ilioinguinal, iliohypogastric and genital nerves. In group A, the ilioinguinal nerve was cut sharply with a blade 1 to 2 cm lateral to the internal inguinal ring, and 3 to 4 cm of the nerve was excised. Neither electrocautery nor suture material was used in cutting the nerve. To control of bleeding, direct pressure was used when needed. After the transection of the nerve, its proximal end was implanted into the internal oblique muscle without tension and drains were not used. A polypropylene mesh was placed as described. We confirmed the division of the ilioinguinal nerve by histopathologic examination.

The patient and the person who completed the forms did not know to which group the patient belonged (study group or control group). The surgeon preserved or excised the nerve according to patient number and the randomization table (double blind). This was not written on the surgery report but could be determined later using the patient number and the randomization table. Severity of pain and hypoesthesia were determined at mentioned endpoints after surgery. VAS was used as a standard scale for rating pain. In addition to pain assessment, numbness was recorded as well. The reliability and validity of this scale among other pain scales has been acceptable. Follow-up was performed by an assessor who was unaware of the procedure and the patients; therefore, the study was conducted in a double-blind fashion. Chi-square and T-Test were used for analysis of data and differences were considered significant at P < 0.05. Collection and analysis of the data was performed using SPSS statistical software version 16.0; all patients were visited at the outpatient clinic at determined endpoints were examined by a physician and a general surgeon who were not aware of the surgical intervention.

## 4. Results

In this study, 140 patients were enrolled ; 66 patients (47.1%) were in nerve excision group and 74 patients (52.9%) were in nerve preserved group; All patients attended the follow up examinations and none of them were excluded at this stage. The mean age of patients was 39.1 ± 11.9 with a range of 18 to 68 years. The mean age in the nerve-excision group was 39.5 ± 13.5 and in the nerve-preserved group was 38.8 ± 10.3; 65 patients were university graduates (46.4%) and the remaining patients (53.6%) were high school graduates. The job of most patients was related to the military forces (44.3%); 19.3% of patients were students, 8.6% were farmers and 12.1% were worker; 84 patients (60%) had right inguinal hernia and 56 patients (40%) had left inguinal hernia. In nerve-excision group 60.4% of patients had right inguinal hernia and 39.4% had left inguinal hernia. Most common complaints were pain and protrusion (both together and concurrently) in the groin area (51.4%); thereafter, protrusion and pain (as a separate complaint) were present in 25.7% and 22.9% of patients respectively.

In the short-term and long-term follow up, there was no significant difference between pain severity after the surgery and chief complaint of patients before surgery. Mean pain severity in patients with pain complaint and in patients with protrusion was 3.16 ± 0.95 and 2.97 ± 1.25, respectively; in patients with both pain and protrusion together, were 2.79 ± 1.12; 22.7% of patients in nerve excision group and 24.3% in nerve preserved group had a background of cardiovascular disease; 110 patients (78.6%) underwent spinal anesthesia and 30 patients (21.4%) underwent general anesthesia.

Overall, the mean incision length was 7.1 cm. The minimum length of incision and maximum length of incision were 5 cm and 10 cm respectively. The average incision length was 7.4 cm in nerve-excision group and 6.9 cm in nerve preserved group. Preoperative mean pain severity on the VAS was 4.48, with the minimum and maximum of 3 and 6. The mean preoperative pain in nerve-excised group was 4.3 and in nerve-preserved group was 4.6. In the short and long term follow ups, there were no significant statistical differences between age and post-surgical pain; mean pain in patients less than 40 years after first week was 2.91 ± 0.99 and in older than 40 years was 2.93 ± 1.28; and mean pain in patients less than 40 years after one year was 0.19±0.39 and in older than 40 years was 0.18 ± 0.39. Mean pain severity one day after surgery in nerve-preserved group and in nerve-excised group was 8.5 ± 0.16 and 7.6 ± 0.7, respectively.

Mean pain severity scores a week after surgery in nerve-preserved group and in nerve-excised group were 3.7 ± 0.7 and 2.03 ± 0.7, respectively. Mean pain severity scores a month after surgery in nerve-preserved group and in nerve-excised group were 1.89±0.6 and 0.56±0.5, respectively. Mean pain severity scores six months after surgery in nerve-preserved group and in nerve-excised group were 0.9±0.6 and 0.3±0.4, respectively. Mean pain severity scores a year after surgery in nerve-preserved group and in nerve-excised group were 0.22 ± 0.44 and 0.15 ± 0.3, respectively. (Table 1)

Mean numbness scores (measured via VAS) a day after surgery in nerve-preserved group and in nerve-excised group were 0.19 ± 0.3 and 0.41 ± 0.4, respectively. Mean numbness scores a week after surgery in nerve-preserved group and in nerve-excised group were 1.3 ± 0.5 and 2.86 ± 0.7, respectively. Mean numbness scores a month after surgery in nerve-preserved group and in nerve-excised group were 1.91 ± 0.7 and 4.61 ± 0.7, respectively. Mean numbness scores six months after surgery in nerve-preserved group and in nerve-excised group were 2.15 ± 0.7 and 5.18 ± 0.7, respectively. Mean numbness scores one year after surgery in nerve-preserved group and in nerveexcised group were 1.3 ± 0.7 and 7.52 ± 0.7, respectively. (Table 2)

Mean return to work time (according to patient’s job) was 5.2 days. Mean return to work time in nerve-preserved group and in nerve-excision group was 5.7 ± 0.9 and 4.6 ± 0.8, respectively. Post-surgical complications in both groups were in Table 3; a day-after surgery. In the first week after surgery six patients had hematoma and seroma; and in six months and one year follow up no problems were found. Infection was seen in 3 patients a month after surgery; tingling in 7 and 6 patients was seen after six months and one year, respectively.

## 5. Discussion

Residual pain should be viewed as an essential endpoint when assessing the outcome of hernia surgery. After 24 to 36 months, nearly 30% of the patients reported some form of pain or discomfort and close to 6% of all patients reported inguinal pain of such intensity that it disturbed their concentration in activities of daily life during the week preceding follow-up (13). The report from the Danish Hernia Data Base Group suggests that the incidence of chronic pain, regardless of grade, 12 months after surgery, is approximately 29%, with 11% of patients complaining of severe pain (3). In a study on the relation between nerve management and CP after open groin hernia repair, the CP rates after 6 months and 5 years were 16.5% and 16.1%, respectively (14). In Caliskan et al. study, one month after operation, the incidence rate of postoperative chronic pain (PCP) was significantly lower in Lichtenstein and neurectomy group versus only Lichtenstein group. At 6 months, there was no significant difference between both groups regarding PCP at rest and coughing. The sensorial changes in the groin region were similar in the two groups (1). In the first month, patients who underwent neurectomy had more inguinal sensory changes than the control group; however, there were no statistically significant differences between two groups 6 months after the operations (1).

In a small randomized controlled trial involving 20 patients with a bilateral inguinal hernia; Ravichandran et al. evaluated the differences in the incidence of pain between the ilioinguinal nerve ‘preserved’ and ‘divided’ sides and did not find any significant difference in the pain and numbness between both sides (6). In another randomized controlled trial; Mui et al. had a lower incidence of chronic pain in the neurectomy group compared to the non-neurectomy group (15). In Sergio et al. study, most of patients with chronic pain slowly recovered at 1 year only with conservative or medical treatment. This suggests that no surgical treatment should be considered for at least 1 year for these patients (3). In a previous prospective study, effectiveness of iliohypogastric neurectomy in preventing postoperative pain was reported; In the consecutive series of 180 anterior tension-free repairs, no patient complained of severe postoperative pain. Two years after surgery, 15 patients complained of hypoesthesia, although no one ever considered it debilitating (9). In a study the incidence of chronic groin pain at 6 months was significantly lower in ilioinguinal neurectomy group than ilioinguinal nerve preservation group. No significant intergroup differences were found regarding the incidence of groin numbness, postoperative sensory loss or changes at the groin region at 6 months after the operation (16). In Malekpour et al. study, post operational pain was less in nerve-excisied group in the first month after surgery. But 6 months and 1 year post-surgery, no significant differences were found (7). Another retrospective study reported that, incidence of chronic groin pain in patients who had elective neurectomy was significantly lower compared with controls. Furthermore, it demonstrated that elective excision of the ilioinguinal nerve was not associated with additional morbidities in neurosensory disturbances, groin numbness or quality of life at the 6-month follow-up (16).

In Ravichandran et al. study, at 6 months postoperatively, pain was present in 1 of 20 patients (5%) on the nervepreserved side versus 0 of 20 patients (0%) on the nerve division side. Numbness was present in 0 of 20 patients (0%) on the nerve-preserved side versus 2 of 20 patients (10%) on the nerve divided side. No significant difference was seen in pain or numbness between the divided and preserved sides. Sensory loss detected by clinical examination was more common following the division of the nerve compared with preservation. Elective division of the ilioinguinal nerve during inguinal hernia repair does not appear to be associated with a significant increase in postoperative symptoms (17). Based on our study, in evaluation of post-surgical complications, no significant differences between two groups were found. This shows that, there is no increase in post-surgical complications after prophylactic excision of the ilioinguinal nerve. Return to work was earlier in the nerve-excised group. In Overall, our study showed that ilioinguinal-excision during Lichtenstein technique significantly decreased post operational pain in short-term follow-up; this demonstrates the superiority of nerve excision surgery. Also it decreases the time to return to work. However, numbness is an inevitable complication, but it seems that painrelief is more important than numbness to patients.

Evaluation of pain was subjective in our study and this is one of the limitations. Another limitation was that the long-term effect of ilioinguinal neurectomy was not investigated. Larger clinical trials with more patients and longer follow-up are warranted to study the long-term effect of prophylactic neurectomy in patients undergoing Lichtenstein repair. And finally we showed that prophylactic neurectomy reduces the incidence of postoperative chronic pain. After the exclusion of patients with preoperative pain the CP rates were 13.2% and 13.8%, in Group A and B, respectively. Our policy was to identify and preserve intact nerves and divide damaged nerves or nerves that were at risk for injury. Ilioinguinal nerve excision at the time of inguinal hernia repair decreased post-surgical inguinal pain and it can be used as a routine method in herniorrhaphy. This procedure is safe and easy to perform during anterior mesh repairs. However, further studies are needed to demonstrate the effectiveness of this procedure in various settings; but this study recommends that Ilioinguinal neurectomy should be considered as a routine surgical step during open mesh hernia repair.
